# Evaluation of the Multifunctionality of Soybean Proteins and Peptides in Immune Cell Models

**DOI:** 10.3390/nu15051220

**Published:** 2023-02-28

**Authors:** Samuel Paterson, Samuel Fernández-Tomé, Alfredo Galvez, Blanca Hernández-Ledesma

**Affiliations:** 1 Department of Bioactivity and Food Analysis, Institute of Food Science Research (CIAL, CSIC-UAM, CEI-UAM + CSIC), Nicolás Cabrera 9, 28049 Madrid, Spain; 2 Department of Nutrition and Food Science, Faculty of Pharmacy, Complutense University of Madrid (UCM), Plaza Ramón y Cajal s/n, 28040 Madrid, Spain; 3 Reliv International, Inc., 136 Chesterfield Industrial Blvd., Chesterfield, MO 63005, USA

**Keywords:** soybean proteins and peptides, lunasin, oxidative stress, immune response, inflammation, biomarkers, cell models

## Abstract

Inflammatory and oxidative processes are tightly regulated by innate and adaptive immune systems, which are involved in the pathology of a diversity of chronic diseases. Soybean peptides, such as lunasin, have emerged as one of the most hopeful food-derived peptides with a positive impact on health. The aim was to study the potential antioxidant and immunomodulatory activity of a lunasin-enriched soybean extract (LES). The protein profile of LES was characterized, and its behavior under simulated gastrointestinal digestion was evaluated. Besides its in vitro radical scavenging capacity, LES and lunasin’s effects on cell viability, phagocytic capacity, oxidative stress, and inflammation-associated biomarkers were investigated in both RAW264.7 macrophages and lymphocytes EL4. Lunasin and other soluble peptides enriched after aqueous solvent extraction partially resisted the action of digestive enzymes, being potentially responsible for the beneficial effects of LES. This extract scavenged radicals, reduced reactive oxygen species (ROS) and exerted immunostimulatory effects, increasing nitric oxide (NO) production, phagocytic activity, and cytokine release in macrophages. Lunasin and LES also exerted dose-dependent immunomodulatory effects on EL4 cell proliferation and cytokine production. The modulatory effects of soybean peptides on both immune cell models suggest their potential protective role against oxidative stress, inflammation, and immune response-associated disorders.

## 1. Introduction

Chronic diseases increased their presence in our society in recent years as a serious disease burden recognized worldwide. With more than 50% of all deaths being attributable to them, inflammation-related diseases such as stroke, cancer, ischemic heart disease, chronic kidney disease, and auto-immune and neurodegenerative conditions are now some of the biggest threats and challenges to human health. Novel studies suggest that the risk of developing chronic inflammation can be traced back to early development in the younger stages of life, and its effects are now known to persist throughout a person’s life to affect adulthood health and the risk of mortality. Moreover, lifestyle factors, including diet, are among the major risk factors contributing to these diseases [1]. Consequently, the development of potential and original preventive and therapeutic strategies against infection, inflammation, and oxidative stress is being done not only to fight these elements directly, but also to complement and reinforce existing therapeutic strategies. Along with diverse pharmaceutical options, bioactive peptides surfaced as one of the most promising strategies [2,3].

Bioactive peptides are defined as inactive amino acid sequences within the source protein that, once liberated after microbial fermentation and the chemical or enzymatic hydrolysis that occurs during food processing or gastrointestinal digestion, can perform different biological activities [4]. Regarding their sources, it should be noted that any food source of protein of animal or vegetal origin can generate bioactive peptides. In the case of vegetable proteins, most studies have focused on cereals and legumes, mainly soybeans [4]. Nowadays, different biological effects of bioactive peptides have been described, such as antioxidant, anticancer or antitumor, immunomodulatory, anti-inflammatory, antidiabetic, antihypertensive, and antimicrobial activity, among others; thus, they exert beneficial effects on the cardiovascular, nervous, and/or immune systems [4,5].

Lunasin, identified 24 years ago, is a soybean peptide encoded by the 2S albumin Gm2S-1 gene [6]. It is made up of the 43-amino acid sequence SKWQHQQDSCRKQLQGVNLTPCEKHIMEKIQGRGDDDDDDDDD (National Center for Biotechnology Information, NCBI, number AAP62458), which has four distinct regions with different functions. Although the soybean is the main source studied for the origin of this peptide, it was also identified in other cereals such as barley, rye, and wheat, although there is still controversy over the results described in the literature [6]. Regarding the bioavailability of lunasin, studies carried out in vitro have delved into its absorption mechanism, and the peptides released after its gastrointestinal digestion were also identified [7]. Thus, it was shown in a monolayer Caco-2 cell model that both lunasin and the ^11^RKQLQGVN^18^ fragment can cross the intestinal epithelium by means of a passive paracellular diffusion mechanism [8,9].

Currently, due to its involvement in the control of various pathologies, the immune system is considered a possible therapeutic target for new strategies against chronic and inflammatory disorders. Plentiful research groups over the past several years have focused their studies on the evaluation of diverse biological activities of the peptide lunasin, so far demonstrating the chemopreventive, antioxidant, anti-inflammatory, hypocholesterolemic, and modulating properties of the nervous and cardiovascular systems, among others [10]. Regarding the immune system, the ex vivo immunomodulatory activity of synthetic lunasin was even explored in primary human cells of the intestinal mucosa [11].

However, most of lunasin’s biological activities, such as its immunomodulatory action, have been carried out with chemically synthesized lunasin [12]. Similarly, lunasin’s potential therapeutic roles will directly depend on its bioavailability and digestibility, which both are related to the type of administration format or the food matrix containing lunasin. The bioactivity of lunasin, and specially its immunomodulatory effects as a part of a food-derived matrix, has not been extensively explored. Therefore, throughout this work, we aimed to study the potential antioxidant and immunomodulatory activities of a lunasin-enriched soybean extract (LES) and to elucidate, in different cell models, its mechanisms of action on biomarkers associated with oxidative stress, inflammation, and immune response.

## 2. Materials and Methods

### 2.1. Materials

2,2′-azino-bis(3-ethylbenzothiazoline-6-sulfonic acid) (ABTS), 6-hydroxy-2,5,7,8-tetramethylchromium-2-carboxylic acid (Trolox), fluorescein disodium (FL), 2,2′-azobis(2-amidinopropane dihydrochloride) (AAPH), 3-(4,5-dimethylthiazol-2-yl)-2,5-diphenyltetrazol bromide (MTT), dimethyl sulfoxide (DMSO), 2′7′-dichlorofluorescein diacetate (DCFA-DA), phorbol 12-myristate 13-acetate (PMA), and ionomycin (Ion) were purchased from Sigma-Aldrich (St. Louis, MO, USA). All other reagents were of analytical grade.

The albumin-enriched soybean extract (ES) was supplied by Reliv International Inc. (Chesterfield, MO, USA). Synthetic lunasin (purity of 95.2%) was obtained by DGpeptides Co., Ltd. (Hangzhou, China).

### 2.2. Preparation of Lunasin-Enriched Soybean Extract (LES)

LES was obtained from ES through the elimination of the insoluble fraction of the sample. First, 500 mg of ES were dissolved in 50 mL of PBS and left stirring at 4 °C overnight. Subsequently, it was centrifuged at 1000× *g* for 5 min, and the supernatant was collected via decantation and then centrifuged two times more under the same conditions. The final supernatant was subsequently lyophilized (LES) and kept at −20 °C until further assays. The protein concentration of LES was measured via the bicinchoninic acid (BCA) method, which was performed using the Pierce BCA kit (Thermo Scientific, Waltham, MA, USA). The standard used was bovine serum albumin (BSA) at concentrations that ranged from 50 to 1000 µg/mL.

### 2.3. Simulated Gastrointestinal Digestion of Lunasin-Enriched Soybean Extract (LES)

The in vitro “INFOGEST” protocol with some modifications was followed to simulate the gastrointestinal digestion of LES [13]. Saliva was collected from five healthy volunteers, pooled, and stored at −80 °C. The enzymes and reagents needed to prepare the simulated gastric fluid (SGF) and the simulated intestinal fluid (SIF) were provided by Merck (St. Louis, MO, USA). A volume of 300 mg of LES was dissolved in 5 mL of saliva, leaving it under mild agitation at 37 °C for 5 min. Next, the pH was adjusted to 3.0 with HCl, 4 mL of the pepsin (EC 3.4.23.1, Sigma-Aldrich, 3026 U/mg) was dissolved in the SGF (1.62 mg/mL), and 2.5 µL of CaCl_2_ was added, incubating the mixture by shaking it at 37 °C for 2 h. Pepsin was inactivated by increasing the pH to 7.0 with NaOH, heating at 85 °C for 15 min, and rapidly cooling the mixture on ice (gastric digest, GD). To carry out the gastrointestinal digestion once the gastric phase was finalized and the pH of the solution was adjusted to 7.0, the bile salts dissolved in Mili-Q water, the CaCl_2_, and the pancreatin (Sigma-Aldrich, 6205 U/mg) dissolved in SIF (76.91 mg/mL) were added, and the mixture was incubated by shaking it at 37 °C for 2 h. The pancreatin was inactivated by heating it to 85 °C for 15 min and rapidly cooling it (gastrointestinal digest, GID). A control of digestion (CD) was also obtained following the same simulated process but in the absence of the sample.

### 2.4. Characterization of the Lunasin-Enriched Soybean Extract (LES) and Its Digests

#### 2.4.1. Gel Electrophoresis (SDS-PAGE)

Sodium dodecyl sulfate polyacrylamide gel electrophoresis (SDS-PAGE) was conducted to evaluate the protein profiles of LES and its digests (GD and GID). Samples dissolved in sample buffer (60 mM Tris-HCl pH 6.8, 25% glycerol (*v*/*v*), 2% SDS (*p*/*v*), 14.4 mM 2-mercaptoethanol, and 0.1% 2-bromophenol (*p*/*v*)) were prepared by heating them at 100 °C for 4 min and then cooling them to room temperature. The samples (50 µg of protein) were loaded into 12% Bis-Tris Criterion^TM^ XT Precast Gel polyacrylamide gel and run through a Mini-Protean Tetra Cell Electrophoresis System (Bio-Rad, Hercules, CA, USA). For separation, the commercial buffer XT MES Running Buffer 20X (Bio-Rad) was used, and the electrophoretic migration and running of the gel were carried out, first with a voltage of 100 V for 5 min and with a voltage of 150 V for 1 h after that. Gel staining was performed with BlueSafe stain (Bio-Rad). Precision Plus Protein Standard Unstained (Bio-Rad) was used as a molecular weight marker. Finally, the gel image was taken using the Versadoc Imaging System (Bio-Rad) gel reader.

#### 2.4.2. Western Blot

The presence of lunasin in LES and its behavior during the simulated digestive process were studied by using a Western blot test. Synthetic lunasin (12.5–50 µM) was employed as the standard. SDS-PAGE was conducted with 16.5% Bis-Tris Criterion™ XT Precast gels (Bio-Rad) and voltage conditions of 60 V for 2 min, which was followed by 100 V for 3 h. Polyvinylidene membranes (PVDF) (Bio-Rad) were used to transfer the proteins by using a voltage of 18 V for 30 min. The membrane was blocked, washed, and incubated overnight (4 °C) with the primary rabbit antibody against lunasin (Biomedal, Seville, Spain, 1:12,000). Then, the incubation of the membrane with the mouse IgG-horseradish peroxidase (HRP)-conjugated anti-rabbit IgG secondary antibody (Santa Cruz Biotechnology, Dallas, TX, USA; 1:5000, 1 h, room temperature) was performed. Finally, the development of the membrane was achieved with the Amersham reagent (Merck, Darmstadt, Germany) for 3 min, and images were taken on the Versadoc Imaging System reader while using an AF 50 mm f/1 4D photographic objective (Nikon, Tokyo, Japan).

### 2.5. Assessment of the Antioxidant Activity of the Lunasin-Enriched Soybean Extract (LES) with Biochemical Assays

The ABTS^•+^ scavenging activity was determined according to the enhanced ABTS^•+^ protocol described by Re et al. [14]. Volumes of 7 mM ABTS and 2.45 mM potassium persulfate were mixed overnight at room temperature to form the ABTS^•+^ radical. Volumes of 180 μL of diluted radical and 20 μL of PBS (blank), Trolox (25–200 µM) (standard), or sample were mixed and incubated for 7 min at room temperature, and absorbance was measured at 734 nm in the Biotek Synergy^TM^ HT plate reader (Winooski, VT, USA). TEAC values are expressed as µmol Trolox equivalents (TE)/mg of protein.

The oxygen radical absorbance capacity (ORAC) was measured following the protocol reported by Hernández-Ledesma and coworkers [15]. Briefly, the mixture (200 µL) containing FL (117 nM), AAPH (14 mM), and either antioxidant (Trolox (0.2–1.6 nmol) or sample) was incubated at 37 °C in 75 mM phosphate buffer (pH 7.4). The fluorescence was recorded every 2 min for 120 min at 485 and 520 nm wavelengths of excitation and emission, respectively, with the Fluostar Optima BMG Labtech plate reader controlled by using FLUOstar Control ver. 1.32 R2 software (Ortenberg, Germany). ORAC values are expressed as µmol TE/mg of protein.

### 2.6. Modulatory Effects in RAW264.7 and EL4 Cell Models

#### 2.6.1. Cell Culture

The mouse RAW264.7 macrophages and lymphoma EL4 cells were purchased from the American Type Culture Collection (ATCC, Rockville, MD, USA) and grown in modified Dulbecco Eagle medium (DMEM) supplemented with 10% of fetal bovine serum (FBS) (*v*/*v*) and 1% penicillin/streptomycin (*v*/*v*) (Biowest, Nuaillé, France). Cells were grown at 37 °C in a humidified incubator containing 5% CO_2_ and 95% air.

#### 2.6.2. Effects on Cell Viability and Cell Proliferation

The evaluation of the effect of LES on the viability of RAW264.7 macrophages was carried out using the MTT assay. Cells were seeded onto 96-well plates (Sarstedt AG & Co., Nümbrecht, Germany) at a density of 6 × 10^4^ cells/well and incubated at 37 °C for 24 h. Then, the culture medium was removed, 120 μL of LES dissolved in DMEM without FBS at different concentrations (0.5–15 μg protein/mL) was added, and the plates were incubated for 8, 16, and 24 h at 37 °C. As negative and positive controls, DMEM medium without FBS and DMEM + lipopolysaccharide (LPS) 10 ng/mL (Merck, Kenilworth, NJ, USA) were used, respectively. After incubation, cells were washed with PBS and incubated with MTT solution (0.5 mg/mL) for 2 h at 37 °C. Once the supernatant was removed, the formazan crystals were dissolved in dimethyl sulfoxide (DMSO), and the absorbance was measured at 570 nm with the Multiskan FC plate reader (Thermo^TM^ Scientific, Wilmington, DE, USA). The results are expressed as percentages of the control, which is considered to be 100%.

The effect of LES on EL4 cell proliferation was evaluated using the WST-1 (Roche, Basilea, Sweden) assay. Cells were seeded onto 96-well suspension plates (Sarstedt AG & Co.) at a density of 5 × 10^3^ cells/well and incubated at 37 °C for 24 h. Then, the cells were treated with LES (0.5–15 μg protein/mL) or synthetic lunasin (1–500 µM) dissolved in DMEM without FBS in the absence or presence of PMA (10 ng/mL) and Ion (100 ng/mL), and they were incubated for 24 h at 37 °C. After that time, the WST-1 reagent was added to the plates, and the cells were incubated for 3 h at 37 °C. The plates were shaken for 1 min at room temperature in the dark, and their absorbance was measured at 450 nm using a Multiskan FC plate reader (Thermo^TM^ Scientific). The cell proliferation rate was calculated relative to that of the control, which was considered to be 100%.

#### 2.6.3. Effects on Reactive Oxygen Species (ROS) Generation

The effect of LES on the intracellular ROS levels was determined following the assay described by LeBel et al. using DCFH-DA as the fluorescent probe [16]. Cells were seeded onto 48-well plates (Corning Costar Corp., Corning, NY, USA) (4.75 × 10^4^ cells/well) and incubated at 37 °C for 24 h. After that time, and once the medium had aspirated, cells were treated with 120 μL of LES dissolved in DMEM without FBS (0.5–15 μg protein/mL), and incubated for 8, 16, and 24 h at 37 °C. The negative and positive controls were DMEM without FBS and DMEM + LPS (100 ng/mL), respectively. After removing the treatment, 100 µL/well of the probe (0.4 mg/mL), which was dissolved in Hank’s Balanced Salt Solution (HBSS, Sigma-Aldrich), was added, and the plate was incubated for 1 h at 37 °C. The fluorescence was measured at λ_excitation_ and λ_emission_ of 485 nm and 520 nm, respectively, by using the Fluostar Optima BMG Labtech plate reader (BMG Labtech). The results are expressed as ROS levels (% compared to the control, which is considered to be 100%).

#### 2.6.4. Effects on Nitric Oxide (NO) Levels

To determine the effect of LES on the release of nitric oxide (NO), the Griess assay was used. Cells were seeded onto 96-well plates (Corning Costar Corp.) at a density of 1 × 10^5^ cells/well and incubated at 37 °C for 24 h. The medium was aspirated, and the cells were treated with 120 μL of LES (0.5–15 μg protein/mL) dissolved in DMEM without FBS for 8, 16, and 24 h at 37 °C. The negative and positive controls were DMEM without FBS and DMEM + LPS (100 ng/mL), respectively. Afterward, 100 µL of the supernatant was mixed with 100 µL of Griess reagent for 15 min at room temperature, and the absorbance was measured at 540 nm in the Biotek Synergy^TM^ HT reader. The amount of NO was calculated using a NaNO_2_ standard curve (3.125–100 μM).

#### 2.6.5. Effects on the Phagocytic Capacity of Macrophages

To study the effect of LES on the phagocytic capacity of the cells, the neutral red assay was used. Macrophages were seeded onto 96-well plates (Sarstedt AG & Co.) at a density of 2 × 10^5^ cells/well and incubated for 24 h at 37 °C. Then, cells were treated with LES (0.5–15 μg protein/mL) for 8, 16, and 24 h at 37 °C. Once the treatment was discarded, a neutral red solution (0.125 mg/mL) was added, and the plate was incubated at 37 °C for 30 min. Lysis buffer (acetic acid 1%:ethanol, 1:1) was added, and the cells were incubated overnight at 37 °C, measuring the absorbance at 540 nm with the Biotek Synergy^TM^ HT plate reader. DMEM without FBS and DMEM + LPS 10 ng/mL were used as negative and positive controls, respectively.

#### 2.6.6. Effects on the Cytokine Release

To evaluate the effect of LES on the cellular production of cytokines (IL-6, IL-1β, IL-10, IL-5, IL-2, IL-4, IL-12 p70, and IFN-ɣ), the corresponding ELISA kits (Mouse IL-6 ELISA Ready-Set-Go!, Mouse IL-1β ELISA Ready-Set-Go!, Mouse IL-10 ELISA Ready-Set-Go, Mouse IL-12 p70 ELISA Ready-Set-Go!, and Mouse IL-5 ELISA Ready-Set-Go! from eBioscience, Affymetrix Company, Santa Clara, CA, USA, and Mouse IFN gamma Uncoated ELISA kit, Mouse IL-4 Uncoated ELISA kit, and Mouse IL-2 Uncoated ELISA kit from Thermo Scientific) were used. Cells were seeded onto 48-well plates (Corning Costar Corp., 1 × 10^6^ cells/well) and incubated at 37 °C for 24 h. Then, cells were treated with LES (0.5–15 μg protein/mL) dissolved in DMEM without FBS. In the case of EL4 cells, PMA (10 ng/mL) and Ion (100 ng/mL) were also added. DMEM medium without FBS was used as a negative control. As positive controls, DMEM + LPS (100 and 1000 ng/mL) and DMEM + PMA (10 ng/mL) + Ion (100 ng/mL) were used for RAW264.7 and EL4 cells, respectively. After 8, 16, and 24 h of incubation at 37 °C of RAW264.7 cells, and after 24 h incubation for EL4 cells, cell supernatants were collected for the ELISA assays by following the manufacturer’s instructions. After being coated with capture antibodies overnight, the plates were blocked, washed, incubated with detection antibodies and horseradish peroxidase-conjugated streptavidin, and finally washed and incubated with the substrate solution. The absorbance was measured at 450 nm using the Multiskan FC plate reader (Thermo^TM^ Scientific), and data were calculated based on the standard curve.

### 2.7. Statistical Analysis

A one-way ANOVA analysis followed by a Bonferroni test was used to analyze the results, using the statistical analysis program GraphPad Prism 7.0 (GraphPad Software, San Diego, CA, USA).

## 3. Results and Discussion

Part of the results shown in this article were presented at the 2nd International Electronic Conference on Nutrients—Nutrition Support for Immunity and Countermeasure Effects on Infection, Inflammation, and Oxidative Stress [17].

### 3.1. Characterization of Lunasin-Enriched Soybean Extract (LES) and Behavior under Simulated Gastrointestinal Digestion

**Figure 1:**
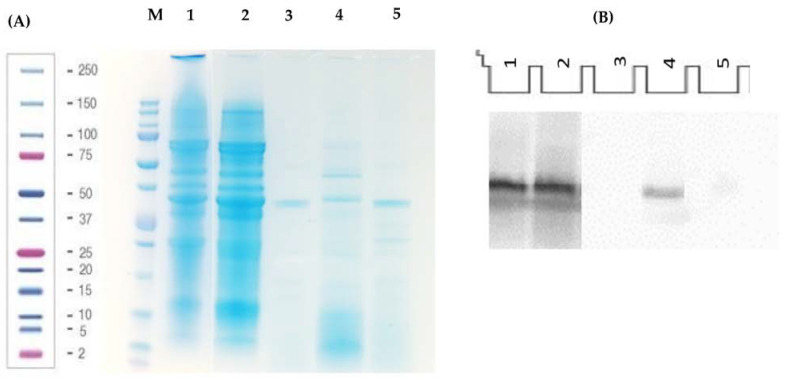
(**A**) SDS-PAGE electrophoresis analysis of (1) albumin-enriched soybean extract (ES, 50 µg protein), (2) lunasin-enriched soybean extract (LES, 50 µg protein), (3) control digest (CD, 50 µg protein), (4) gastric digest from LES (GD, 50 µg protein), and (5) gastrointestinal digest from LES (GID, 50 µg protein). M: Precision Plus Protein Standard Unstained molecular weight marker. (**B**) Analysis of LES and its gastric and gastrointestinal digests by Western blot assay in PVDF membrane. (1) Synthetic lunasin (12.5 µM), (2) LES (50 µg protein), (3) control digest (CD, 50 µg protein), (4) gastric digest from LES (GD, 50 µg protein), and (5) gastrointestinal digest from LES (GID, 50 µg protein).

The protein concentration of ES was 52.7% and decreased up to 12.6% when aqueous solvent extraction was carried out to remove insoluble proteins and recover LES. The protein profiles of both samples were analyzed via SDS-PAGE, as shown in Figure 1A. Both profiles were similar, with bands corresponding to proteins with molecular weights ranging from 4 to 138 kDa. However, the intensities of some bands in LES were higher than those shown by ES, indicating the enrichment of these proteins after extraction with the aqueous solvent. It was possible to observe the major soybean proteins, glycinin, β-conglycinin and its corresponding α and α’ subunits (82.03 kDa and 76.14 kDa, respectively), and the subunits glycinin A1a and A2 (36.11 kDa), which similarly correspond to the molecular weights previously described for these proteins by different authors [18].

The presence of peptide lunasin in LES was detected in the Western blot assay by using an antibody specific for this peptide (Figure 1B). The concentration, which was quantified by using a standard curve with synthetic lunasin (y = 11,099x; R^2^ = 0.9922), was 16.42 mg lunasin/g protein, or 2.07 mg lunasin/g of extract. This concentration was lower compared to that reported for a lunasin-enriched soy extract produced in a two-step pilot plant-based ultrafiltration process (58.2 mg lunasin/g protein) [19], although it was within the reported range for different lunasin-based commercial soybean products (9.2 ± 0.6 to 25.7 ± 1.1 mg lunasin/g protein) [20,21].

The behavior of the proteins and peptides contained in LES during gastrointestinal digestion was studied by using the SDS-PAGE and Western blot tests. The electrophoretic analysis revealed the susceptibility of high molecular weight proteins to the action of pepsin during the gastric phase of digestion, releasing proteins and polypeptides with lower molecular weights that were further digested by pancreatic enzymes. However, some proteins showed resistance to the action of both gastric and intestinal digestion, as their bands appeared at the end of the digestive process (Figure 1A). It was possible to identify the presence of lunasin after the gastric phase, and 60% of the initial lunasin remained in the GD. However, pancreatic enzymes degraded lunasin to a greater extent, and only 2.9% of the intact peptide could be visualized in the gastrointestinal digest (GID). Previous studies with synthetic lunasin described the susceptibility of the peptide to the action of digestive enzymes, which was partially reversed by the presence of protease inhibitors such as the Bowman–Birk inhibitor (BBI) or the Kunitz inhibitor [21,22]. The inhibitors potentially inherent in LES could, therefore, be those responsible for the resistance of lunasin to the digestive process. The residual lunasin at the end of the digestive process could exert its effects locally, or it could be absorbed and reach the target organs. In fact, lunasin was identified in human plasma samples after soy protein consumption [23].

### 3.2. Antioxidant Activity of Lunasin-Enriched Soybean Extract (LES) and Its Digests

The antioxidant activity of ES and LES was evaluated with the biochemical assays ORAC and ABTS, and higher effects for LES (1.67 μmol TE/mg protein and 0.23 μmol TE/mg protein, respectively) than for ES (0.50 μmol TE/mg protein and 0.11 μmol TE/mg protein, respectively) were observed. The enrichment in low-molecular weight peptides during LES preparation could favor the increase of antioxidant potential. Thus, peptides containing between 5 and 16 amino acids were identified and characterized as the major factors responsible for the antioxidant effects of soybean fractions [12,24,25,26]. The TEAC value of LES (28.97 μmol TE/g LES) was slightly lower than those observed in other soybean samples (44.9–50.0 μmol TE/g of extract) [27,28]. These differences could be due to its soybean origin as well as to the treatment and extraction conditions used in each study. In addition, the effect of simulated gastrointestinal digestion on the radical scavenging capacity of LES was evaluated. It was found that after the action of gastric and pancreatic enzymes on LES, its antioxidant activity increased up to 2.17 µmol TE/mg protein (ORAC) and 0.33 µmol TE/mg protein (TEAC) in GID. These findings indicate that fragments released from lunasin or from other proteins contained in LES could exert more potent antioxidant effects than their protein source. Similarly, the peroxyl radical scavenging capacity of soybean concentrates and of corolase PP hydrolyzates notably increased after being digested, simulating gastrointestinal conditions [29,30]. Peptides released during the digestion process could be responsible for this increase in the radical scavenging capacity of gastrointestinal digests.

### 3.3. Effects of Lunasin-Enriched Soybean Extract (LES) in RAW264.7 Macrophages and EL4 Cells

To evaluate the action of LES on biomarkers associated with oxidative stress, inflammation, and immune response, two immune cell models, RAW264.7 macrophages and lymphocytes EL4, were used. First, the dose- and time-dependent effects of LES on the viability of RAW264.7 macrophages cells were evaluated with the MTT assay. Cells were treated with concentrations that ranged from 0.5 to 15 µg protein/mL for 8, 16, and 24 h. In Table 1, the percentages of viable cells after 8 and 24 h of treatment with LES or LPS are shown.

After 8 h, LES significantly increased the viability of macrophages, whereas LPS (10 ng/mL) did not provoke any significant effect. This result indicates an immunostimulatory effect of protein and peptides contained in LES at short incubation times. However, when the cells were incubated for 16 h (data not shown) and 24 h (Table 1), LES inhibited cell viability at concentrations higher than 7.5 µg protein/mL and 2.5 µg protein/mL, respectively. After 24 h of treatment, the highest assayed dose of LES (15 µg protein/mL) inhibited cell viability up to 47.0%, whereas LPS reduced viability by 23.8%. This result indicates that both protein and peptides contained in LES exerted cytotoxic effects on macrophages, as previously reported for soybean protein-derived peptides [31].

To determine whether peptides contained in LES could scavenge the ROS, a DCFH-DA assay was carried out. As shown in Table 1, LPS treatment at 100 ng/mL induced greater ROS accumulation compared to untreated cells at the three times tested. The highest induction was observed after 8 h of stimulation, reaching ROS levels of 124.9 ± 12.6% compared to non-induced cells (100.0 ± 10.0%). These ROS-inducing effects were previously reported in RAW264.7 cells treated with LPS at 2 µg/mL for 24 h [32]. In the case of LES, it exerted protective effects against oxidative stress at the three assayed times, reducing ROS levels at doses lower than 7.5 µg protein/mL. The radical scavenging activity demonstrated in this study with biochemical assays could contribute to the ROS-reducing effects of LES. A previous study carried out in our laboratory demonstrated that synthetic lunasin exerted potent protective effects on cell viability and oxidative status in RAW264.7 macrophages challenged with chemicals tert-butylhydroperoxide and hydrogen peroxide [22]. Recent findings show that liposomes loaded with amaranth unsaponifiable matter and soybean lunasin (0.5–2 mg/mL) have an antioxidant effect in RAW264.7 macrophages by significantly decreasing ROS production after 1 h of treatment [33]. The protective effects of lunasin against oxidative stress was also demonstrated in other cell lines. Thus, pepsin-pancreatin hydrolyzates from a lunasin-enriched preparation were shown to inhibit the production of intracellular ROS in THP-1 human macrophages [34]. In challenged Caco-2 cells, synthetic lunasin (0.5–10 µM) also exerted a protective role by reducing the ROS increase induced by chemical agents [35]. Thus, the lunasin present in LES could be responsible for its antioxidant effects, although other soybean derived peptides could also provide important contributions. At the highest assayed dose of LES, a significant pro-oxidant effect was observed when cells were treated for 24 h (Table 1). Even though the increase of intracellular ROS could favor a more efficient destruction of microorganisms during the immune response of the macrophages [36], the oxidizing effect of LES at the highest concentration should be explored more in detail to understand the balance between antioxidant and oxidant compounds present in LES and their influence in the redox metabolism of immunological cells such as macrophages.

**Figure 2:**
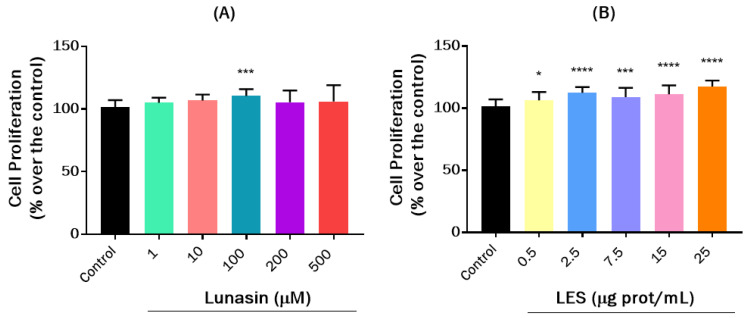
Effects of lunasin and lunasin-enriched soybean extract (LES) on the proliferation of EL4 cells. (**A**) Effects of synthetic lunasin (1–500 µM) on the cell proliferation (% over the control) of EL-4 cells under basal conditions after 24 h of treatment. (**B**) Effects of LES (0.5–25 µg protein/mL) on EL4 cell proliferation (% over the control) under basal conditions after 24 h of treatment. Significant differences compared to control: * *p* < 0.05; *** *p* < 0.001; **** *p* < 0.0001.

To further investigate the immunomodulatory effects of lunasin and other soybean peptides contained in LES on cells related to the adaptative immune system, a lymphocyte EL4 model was also explored. The effects of LES (0.5–25 μg protein/mL) and lunasin (1–500 μM) on cell proliferation after 24 h of treatment under basal conditions are shown in Figure 2. Previous findings show that synthetic lunasin stimulates EL4 proliferation irrespective of the presence or absence of obesity-inducing factors [37]. However, in our study, lunasin did not cause any effect on cell proliferation (Figure 2A). In the case of LES, a significant dose-dependent increase of EL4 cell proliferation was observed (Figure 2B), suggesting that other peptides could be responsible for the immunostimulant effects of LES. The effects of lunasin and LES on EL4 cells challenged by PMA and Ion were also evaluated. PMA and Ion are common reagents used in immune cell culture for their capacity to activate T cells, increasing their proliferation and stimulating the release of different cytokines [38]. In our study, the increase in EL4 cell proliferation caused by the combination of PMA and Ion was not reversed with LES or lunasin (data not shown).

**Figure 3:**
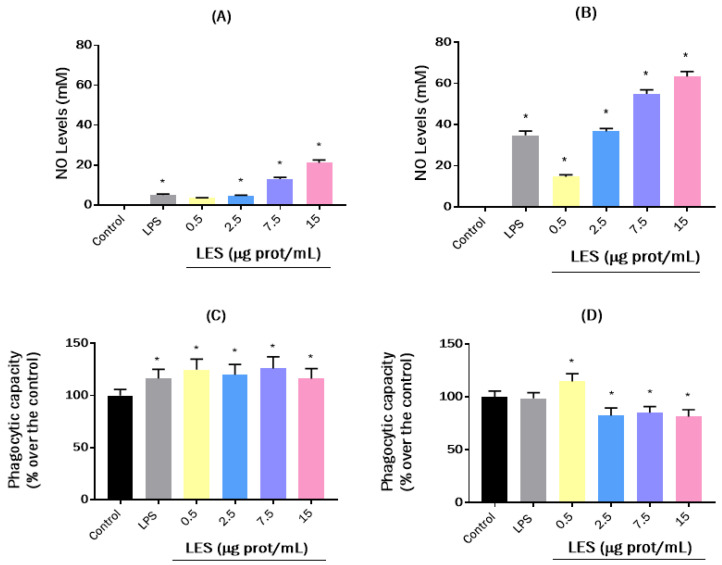
Immunomodulatory effects of lunasin-enriched soybean extract (LES) on RAW264.7 macrophages. (**A**,**B**) Effects of lunasin-enriched extract (LES, 0.5–15 µg protein/mL) on nitric oxide (NO) levels in RAW264.7 cells after (**A**) 8 h and (**B**) 24 h of treatment. (**C**,**D**) Effects of LES (0.5–15 µg protein/mL) on the phagocytic capacity of RAW264.7 cells after (**C**) 8 h and (**D**) 24 h of treatment. LPS was used as a positive control at a concentration of (**A**,**B**) 100 ng/mL for NO induction and (**C**,**D**) 10 ng/mL for phagocytic capacity stimulation. Significant differences compared to control: * *p* < 0.0001.

The effects of LES on NO production by RAW264.7 cells were assessed by using the Griess assay. NO is a molecule that acts as an inflammation biomarker, as it is harmful to the body at high concentrations [39]. However, macrophages produce this compound as an intermediary of their cytotoxic action against pathogens, favoring their phagocytic activity [40]. Although NO production could not be detected at basal conditions, after stimulating the cells with LPS, NO levels significantly increased in a time-dependent manner, reaching values of 4.96, 17.47, and 34.81 µM at 8, 16, and 24 h, respectively (Figure 3A,B). LES also caused a stimulating effect on NO production in a dose- and time-dependent manner. Hence, at the maximum LES concentration tested, the NO levels detected were 21.08, 43.19, and 63.40 μM at 8, 16, and 24 h of treatment, respectively. Soybean lunasin at 10 µM was reported to inhibit NO production induced by LPS in macrophages [41], as it had been demonstrated for quinoa-derived lunasin at a dose of 0.40 g/L [42]. This controversy could be due to the concentration of lunasin in LES or to the presence of other peptides able to induce NO release. Similarly, peptides from other food sources were also found to induce NO production in macrophages. For example, bioactive peptides from European eel *Anguilla anguilla* stimulated NO release in a dose-dependent manner when basal RAW264.7 cells were treated for 24 h [43]. The treatment of macrophages for 24 h with the peptide YGPSSYGYG from *Pseudostellaria heterophylla* protein hydrolyzates also resulted in cell activation and an increase of NO levels [44]. The results obtained in this work suggest that the immunostimulatory effect of LES in macrophages, by increasing their NO production, could favor their cytostatic and cytotoxic capacity and, on the other hand, exert a vasodilator effect that would help the other cells of the immune system reach the possible source of infection. To elucidate the effect of LES on the phagocytic capacity of RAW264.7 macrophages, the neutral red assay was carried out. The results obtained after 8 and 24 h of treatment are shown in Figure 3C,D. After 8 h, phagocytic capacity increased significantly at the four concentrations tested without differences among them. The stimulatory effect was similar to that observed for cells induced with LPS (Figure 3C). Previous findings reported the immune-enhancing effects of food-derived proteins and peptides by increasing the phagocytic capacity of macrophages [45]. After 16 h (data not shown), phagocytic capacity was affected by neither LPS nor LES. Finally, at 24 h, LPS did not modify the phagocytic capacity of the cells. In the case of LES, only the lower concentration caused an increase in phagocytic capacity, whereas the higher concentrations caused a significant decrease in phagocytosis, which could be due to the inhibitory effect of cell viability exerted by the high concentrations of the extract after 24 h of incubation (Figure 3D).

Cytokines are soluble proteins secreted from a variety of cells (lymphocyte, macrophage, natural killer, mast, and stromal cells) that can act as mediators of inflammation and immune responses [46]. Given their key role in the immune system, the effects of LES on the secretion of IL-6, IL-1β, IL-10, IL-12 p70, and IFN-ɣ by RAW264.7 macrophages were evaluated. IL-12 p70 could not be detected in the cell supernatants obtained after treatment with LES.

**Figure 4:**
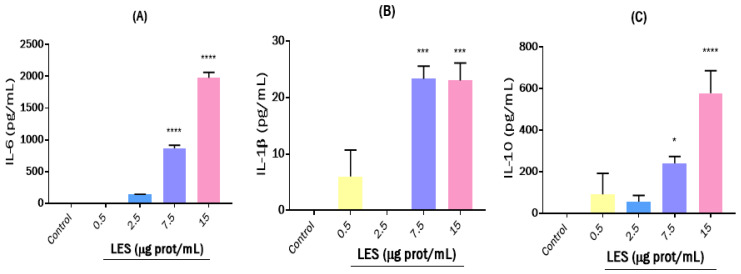
Effects of lunasin-enriched soybean extract (LES) on cytokine release by RAW264.7 macrophages. Effects of LES (0.5–15 µg protein/mL) on the release of (**A**) IL-6, (**B**) IL-1β, and (**C**) IL-10 after 8 h of treatment. Significant differences compared to control: * *p* < 0.05; *** *p* < 0.001; **** *p* < 0.0001.

In the case of IFN-γ, only the highest concentration of LES was able to slightly stimulate this cytokine after 24 h of treatment (data not shown). IFN-ɣ primarily mediates antiviral and antibacterial immunity, enhancing antigen presentation and the innate immune system activation that results in a Th1 proinflammatory response [47]. Our previous study demonstrated the ability of synthetic lunasin to abrogate the ex vivo secretion of IFN-γ in human intestinal biopsies [11]. In Figure 4, the effects of LES on the secretion of IL-6, IL-1β, and IL-10 after 8 h of treatment are shown.

As shown in Figure 4A, LES caused a dose-dependent stimulatory effect of IL-6 secretion until reaching a value of 1978 pg/mL at the highest dose assayed (15 µg protein/mL) after 8 h of treatment. The stimulation was more notable when cells were treated for 16 h (data not shown), and IL-6 levels were 5190 pg/mL, which was slightly lower than those measured in the supernatants obtained from LPS-challenged cells (6023 pg/mL). Although IL-6 is mostly considered as a proinflammatory cytokine, it also performs many regenerative or anti-inflammatory activities [48]. Thus, in models of chronic inflammatory diseases, such as collagen-induced arthritis, murine colitis, or experimental autoimmune encephalomyelitis, IL-6 acts as proinflammatory cytokine, whereas in models of acute inflammation, it also exhibits an anti-inflammatory profile [49].

LES also showed a dose-dependent stimulatory effect on IL-1β secretion at the three times assayed, reaching values for this cytokine up to 23.04, 12.54, and 3.81 pg/mL after 8, 16 (data not shown), and 24 h of treatment (Figure 4B), respectively, at the dose of 15 µg protein/mL. These induced effects were more potent than those resulting from LPS. IL-1β is a member of the IL-1 family of cytokines produced by activated macrophages and recognized as an important mediator of the inflammatory response, as it is involved in a variety of cellular activities, including cell proliferation, differentiation, and apoptosis [50]. Other proteins and peptides were also found to induce IL-1β secretion in immune cell models. For example, lactoferrin and zinc-supplemented lactoferrin exerted stimulating dose-dependent effects on IL-1β secretion in RAW264.7 macrophages after 24 h of incubation [51]. However, lunasin was reported to reduce IL-1β secretion in RAW264.7 cells cultured in both adipocyte-conditioned medium and leptin-containing medium [52]. Thus, the low concentration of lunasin in LES or the presence of other peptides with stimulatory properties on IL-1β release that surpass the downregulatory properties of lunasin could be determined by the observed effects for the soybean extract.

LPS (1000 ng/mL) had a stimulatory effect on IL-10 levels that decreased over time, with values of 2366.8 pg/mL, 1966.1 pg/mL, and 335.7 pg/mL for cells treated for 8, 16, and 24 h (Figure 4C), respectively. When cells were treated with LES at 0.5 and 2.5 μg protein/mL, a slight but not significant stimulating effect was observed. However, this effect was more potent when LES was used at higher concentrations, reaching IL-10 values of 240.03 pg/mL and 577.24 pg/mL for 7.5 and 15 μg protein/mL doses, respectively, after 8 h of treatment (Figure 4C), and 642.2 pg/mL and 956.0 pg/mL for 7.5 and 15 μg protein/mL doses, respectively, after 16 h (data not shown). These values were diminished after 24 h of treatment. IL-10 is a cytokine with potent anti-inflammatory action produced by T cells, B cells, macrophages, and keratinocytes, and it can profoundly alter cell morphology and the production of cytokines by monocytes, which in turn can affect a variety of immunological responses [53]. Therefore, IL-10 plays a central role in limiting the host’s immune response to pathogens, thereby preventing damage to the host and maintaining normal tissue homeostasis [54]. Controversial results on the effects of soy and derived compounds can be found in the literature. For example, some studies have reported the IL-10-inducing effects of soy isoflavones in RAW264.7 macrophages [55], whereas others showed no significant effects [56,57,58]. In the case of soybean polypeptides, the BBI demonstrated that it can regulate inflammation by increasing anti-inflammatory IL-10 levels in LPS-stimulated RAW264.7 and other immune cells [59]. Therefore, the observed stimulatory effects of LES on IL-10 production cells could be the result of the action of multiple compounds. The combined action of LES on the cytokine secretion, NO release, and phagocytic capacity of macrophages suggests the potential action of this soybean extract to activate immune cells as antigen-presenting cells, pathogen detectors, and initiators of the inflammatory innate immune response.

**Figure 5:**
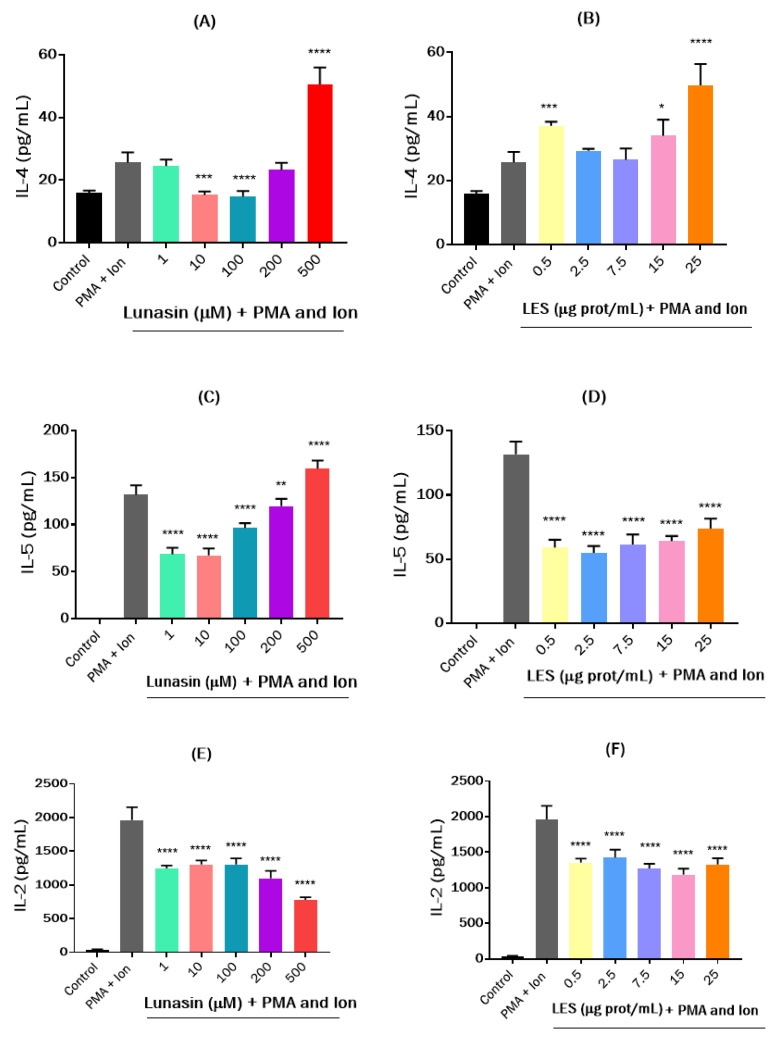

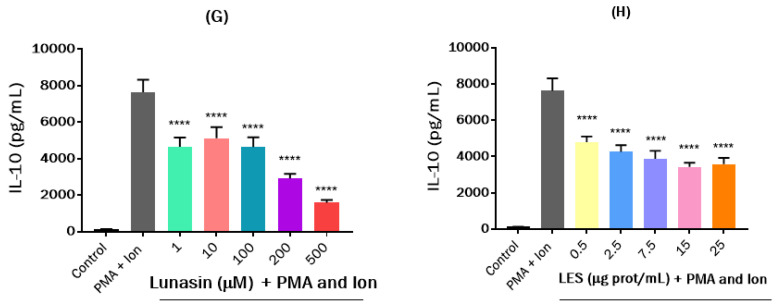
Effects of synthetic lunasin and lunasin-enriched soybean extract (LES) on cytokine release by stimulated EL-4 cells. Effects on the release of (**A**,**B**) IL-4, (**C**,**D**) IL-5, (**E**,**F**) IL-2, and (**G**,**H**) Il-10 of (**A**,**C**,**E**,**G**) synthetic lunasin (1–500 µM) and (**B**,**D**,**F**,**H**) LES (0.5–15 µg protein/mL) by EL-4 cells induced by phorbol 12-myristate 13-acetate (PMA, 10 ng/mL) and Ionomycine (Ion, 100 ng/mL) after 24 h of treatment. Significant differences compared to PMA + Ion-induced cells: * *p* < 0.05; ** *p* < 0.01; *** *p* < 0.001; **** *p* < 0.0001.

To study the immunomodulatory activity of lunasin and LES in the adaptive immune system, their effects on the production of cytokines IL-4, IL-5, IL-2, and IL-10 were evaluated in PMA-Ion challenged EL4 cells after 24 h of treatment. Doses of LES ranged from 0.5 to 25 μg protein/mL, and doses of lunasin ranged between 1 to 500 μM. The results are shown in Figure 5. Lunasin, at intermediate doses of 10 and 100 μM, significantly reverted the inducing effects of the PMA and Ion combination on IL-4 production, whereas the highest dose provoked a notable challenge to cells, increasing IL-4 values up to 50.60 pg/mL (Figure 5A). LES, at all tested doses, potentiated the inducing effects of PMA and Ion (Figure 5B). IL-4 is a cytokine produced primarily by mast cells, Th2 cells, eosinophils, and basophils that has many biological roles, including the stimulation of activated B cell and T cell proliferation, the differentiation of B cells into plasma cells, and the differentiation of naive helper T cells to Th2 cells [60]. Plant-derived bioactive compounds show different effects on IL-4 production. Thus, phytoestrogen formononetin and its metabolites, daidzein and equol, were found to significantly enhance IL-4 production in both CD4^+^ T cells and EL4 cells in a dose-dependent manner [61], whereas the pigment shikonin and its derivatives from the Chinese medicinal herb *Lithospermum erythrorhizon* showed inhibitory effects on mitogen-induced IL-4 and IL-5 production in EL4T cells [62]. All lunasin doses but 500 μM reduced increased IL-5 production caused by PMA and Ion (Figure 5C). LES also had a significant inhibitory effect for all doses assayed, reaching 73.8 pg/mL in supernatants collected from cells treated with 25 μg protein/mL of LES compared to the PMA + Ion group (131.8 pg/mL, Figure 5D). IL-5 is mainly produced by type-2 T helper cells and mast cells, and it acts by stimulating B cell growth and increasing immunoglobulin (Ig) secretion, primarily IgA. It is also a key mediator in eosinophil activation and is associated with the etiology of several allergic diseases, including allergic rhinitis and asthma [63,64]. Comparably to LES, previous studies with other plant-based bioactive compounds showed that they display a similar downregulatory effect [65,66].

IL-2 is a proinflammatory cytokine mainly secreted by activated CD4^+^ and CD8^+^ T cells that plays a pivotal role in both regulatory T cell biology and immune response homeostasis between tolerance and immunity [67,68]. In comparison with PMA + Ion treated cells (1963.7 pg/mL), both lunasin and LES presented a significant inhibitory effect on IL-2 secretion. In the case of synthetic lunasin, the effects were dose-dependent, reaching a value of 776.9 pg/mL in the supernatant collected from cells treated with 500 µM of peptide (Figure 5E). However, a recent study found that lunasin, at concentrations of 10 and 50 µM, was able to significantly increase IL-2 secretion in PMA + Ion-challenged EL4 cells under conditions simulating an obesity-related microenvironment [37]. In contrast, although LES also reverted the IL-2-inducing effects of the PMA and Ion combination, no dose-dependence was observed, with IL-2 values ranging from 1186.5 to 1425.04 pg/mL (Figure 5F). However, the protective effects against the increase of IL-10 induced by PMA + Ion were dose-dependent for both LES and synthetic lunasin. Individual peptides showed stronger inhibitory activity, as the measured value of this cytokine in the supernatant collected from the lunasin (500 µM)-treated group was 1586.5 pg/mL, almost five times lower than the value measured in the PMA + Ion group (7640.7 pg/mL) (Figure 5G). These results are different from those found by Hsieh et al. that did not report any effect on IL-10 secretion by PMA + Ion activated EL4 cells under simulated obese conditions [37]. These differences could be due to the absence of obesity-related conditions in our assay that modified the response of cells to lunasin treatment. In the case of the supernatant collected from the LES-treated group, the measured IL-10 level was 3586.8 pg/mL (Figure 5H). Similarly, other bioactive compounds from food sources were also found to suppress IL-2 production by EL4 cells [69].

## 4. Conclusions

The enrichment in soluble proteins and small peptides such as lunasin was achieved during LES preparation. Among them, the protease inhibitors could be responsible for the protection against the action of digestive enzymes on lunasin, favoring its beneficial effects at both local and systemic levels. The in vitro radical scavenging action of LES was confirmed in the RAW264.7 macrophages model, observing a protective role on oxidative stress at low doses. Moreover, LES was found to exert immunostimulatory effects in both innate (RAW264.7) and adaptive (EL4) immune cell models. A dose- and time-dependent immunostimulant effect was observed in RAW264.7 macrophages in terms of their phagocytosis activity as well as in terms of NO, IL-6, IL-1β, and IL-10 production. Likewise, lunasin and LES exerted a dose-dependent immunomodulatory effect on EL4 cell proliferation and cytokine production. The effects of lunasin and soybean protein-based samples on macrophage phagocytosis specifically are still a scattered topic; nevertheless, these findings in combination with the increase in NO levels and regulatory cytokines mentioned before suggest that LES immunomodulatory action could lean towards the stimulation of macrophages; thus, it would favor their cytotoxic action and ability to eliminate pathogens. EL4 activation and cytokine production could potentially indicate that soybean protein’s immunomodulatory effects do not end in the innate immune system but continue to modulate the adaptive immune cells’ activity. Further research is needed to confirm this hypothesis and to identify the contribution of each protein/peptide in order to confirm the potentiality of this extract as an ingredient of functional foods and supplements with beneficial effects on immune response.

## Figures and Tables

**Table 1:** Effects of lunasin-enriched soybean extract (LES) on RAW264.7 cell viability and reactive oxygen species (ROS) production (% over the control) after 8 h and 24 h of treatment.

Condition	Viable Cells (% over Control)		ROS (% over Control)	
	8 h	24 h	8 h	24 h
Control	100.0 ± 5.6	100.0 ± 4.2	100.0 ± 10.0	100.0 ± 6.2
LPS ^a^	107.7 ± 9.2	76.2 ± 8.4 ****	124.9 ± 12.6 ****	115.2 ± 7.4 *
LES 0.5 µg protein/mL	116.4 ± 12.6 ****	105.4 ± 6.3	74.4 ± 6.1 ****	81.6 ± 6.9 **
LES 2.5 µg protein/mL	118.5 ± 12.9 ****	83.6 ± 5.4 **	73.5 ± 8.6 ****	77.8 ± 6.2 ***
LES 7.5 µg protein/mL	118.4 ± 12.7 ****	65.2 ± 7.7 ****	74.5 ± 0.9 ****	76.5 ± 5.3 ***
LES 15 µg protein/mL	119.2 ± 6.5 ****	53.0 ± 7.2 ****	105.0 ± 8.9	136.1 ± 12.5 ****

Significant differences compared to control (* *p* < 0.05; ** *p* < 0.01; *** *p* < 0.001; **** *p* < 0.0001). ^a^ LPS was used at concentrations of 10 ng/mL and 100 ng/mL in the cell viability and reactive oxygen species (ROS) production assays, respectively.

## Data Availability

Not applicable.
